# Short-Term Prognostic Value of the Culprit-SYNTAX Score in Patients with Acute Myocardial Infarction

**DOI:** 10.3390/jcdd10070270

**Published:** 2023-06-24

**Authors:** Tezcan Peker, Bedrettin Boyraz

**Affiliations:** Cardiology Department, Health Sciences Faculty, Medicalpark Hospital, Mudanya University, Bursa 16200, Turkey; bedrettin.boyraz@mudanya.edu.tr

**Keywords:** ST-elevation myocardial infarction, non-ST-elevation myocardial infarction, percutaneous coronary intervention, SYNTAX score, culprit-SYNTAX score

## Abstract

Background: The SYNergy between Percutaneous Coronary Intervention with TAXus and Cardiac Surgery (SYNTAX) score is a scoring system that helps to decide on surgery or percutaneous coronary intervention (PCI) in patients with acute myocardial infarction (MI), and studies are showing the prognostic value of this scoring system in both AMI and coronary artery disease patients undergoing PCI. In acute coronary syndrome (ACS) patients, the infarct-related artery and the complexity of the lesions are also important in terms of mortality and morbidity. Our study aimed to determine the prognostic value of the culprit vessel’s SYNTAX score (cul-SS) in patients presenting with MI. Methods: In our study, 1284 patients presenting with MI were analyzed retrospectively. The SYNTAX scores and cul-SS of the patients were calculated. In-hospital and 30-day deaths and major complications were accepted as primary outcomes. The SYNTAX scores and cul-SS were compared in terms of predicting primary outcomes. Conclusions: Major complications were observed in 36 (2.8%) patients, death in 42 (3.3%) patients, and stent thrombosis in 24 (1.9%) patients. The area under the curves for SYNTAX and cul-SS for predicting primary outcomes is 0.64 and 0.68 (*p* = 0.026), respectively. Cul-SS was as successful as the SYNTAX score in predicting stent thrombosis and was superior in predicting short-term death and major complications.

## 1. Introduction

Short-term mortality and major complication rates are higher in patients admitted to the hospital due to acute coronary syndrome (ACS) compared to patients admitted to the hospital with chronic coronary artery disease (CAD) and undergoing intervention. The main difference here is due to myocardial dysfunction caused by an occluded coronary artery, susceptibility to arrhythmias, and hemodynamic instability. In-hospital treatment modalities may also differ between ACS and non-ACS patients. While eliminating the ischemic condition as soon as possible and mostly avoiding early complications is the first priority in ACS patients, the first priority in non-ACS patients is to achieve long-term success. Percutaneous coronary intervention (PCI) is often a priority in ACS patients due to the clinical condition of the patients and the lesions in their coronary vessels. In chronic CAD patients, the decision for PCI and surgical treatment is more often made based on the “Synergy between Percutaneous Coronary Intervention with Taxus and Cardiac Surgery” (SYNTAX) score compared to ACS patients. The SYNTAX score is an anatomical scoring system that was developed to show the extent of CAD angiographically and to decide on the choice of revascularization method [1,2,3,4]. Parameters such as the vessel in which the lesion is located, the number of lesions, the presence of the lesion in the proximal or distal regions of the vessel, and whether it is a bifurcation or chronic total occlusion (CTO) lesion are used to calculate the SYNTAX score [5,6,7]. In addition, it is valuable and widely used in demonstrating short- and long-term prognosis in patients presenting with acute myocardial infarction (MI). It has been shown that the SYNTAX score can predict cardiac death, MI, and target lesion revascularization (TLR) in both patients with stable CAD and ACS [8,9,10,11,12,13,14].

In ACS patients, the infarct-related artery and the complexity of the lesion are also important in terms of mortality and morbidity. Lesions in the left main coronary artery (LMCA) or equivalent lesions can cause a serious increase in both mortality and morbidity. In addition, proximal lesions of the left anterior descending artery (LAD) are of serious importance for patients both in the short and long term, as they provide blood supply to a high amount of myocardial tissue. It is known that the region fed by the infarct-related artery and the proximal location of the lesion increase mortality in acute MI patients [15,16]. In addition, the complexity of the lesion and its proximal location can influence whether PCI is applied to complex patients. While CTO lesions pose a risk in terms of technical difficulty, bifurcation lesions may require higher levels of experience. In addition, the presence of diffuse vascular disease or calcific lesions makes it difficult to provide adequate flow and stent apposition, while lesions with dense thrombus may cause coronary perfusion to not be achieved at the desired level. This and similar situations can both prolong the duration of the procedure and increase the possibility of complications.

There is no clear, routine scoring system that can be applied to evaluate the responsible lesion from all aspects. Frequently, the approach is based on descriptions such as LMCA and LAD proximal, and no scoring system is used to describe the risk of lesions. No scoring system defines the severity of the lesion in the infarct-related artery and evaluates it in terms of clinical outcomes in patients presenting with ACS. While the widely used GRACE risk score assigns a risk score according to the clinical condition of the patients, the SYNTAX risk score evaluates the total coronary plaque burden of the patients. However, although total coronary plaque burden is effective for short- and long-term prognosis in ACS patients, the infarct-related artery is more important in terms of prognosis in the short term. Based on these data, we based our study hypothesis on the fact that the short-term prognosis depends more on the plaque load of the culprit lesion and the size of the myocardial tissue at risk due to MI than the total coronary plaque load. The relationship between the culprit lesion’s SYNTAX score (cul-SS) and the SYNTAX score was investigated and compared in terms of short-term death, major complication rates, and stent thrombosis. Based on the results of the study, we tried to obtain a scoring system that could show the short-term prognosis of the patients based on the SYNTAX score of the culprit lesion.

## 2. Materials and Methods

**Figure 1:**
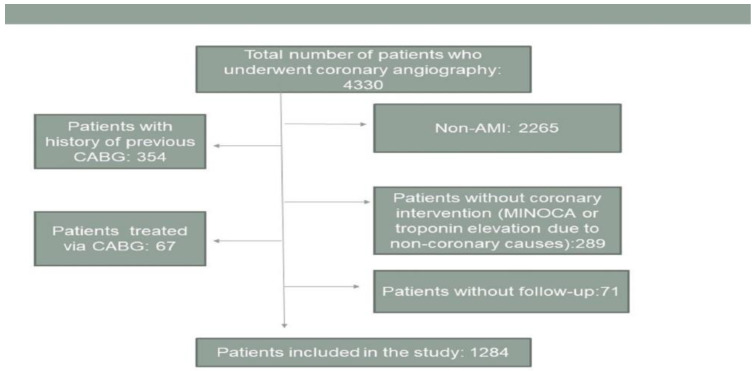
Patient selection.

All study procedures involving human participants were in accordance with the ethical standards of the institutional and national research committees and with the 1964 Helsinki Declaration and its later amendments or comparable ethical standards. The study was approved by the local Ethical Committee. This study is designed as a retrospective, single-centered study. The study included 1284 patients admitted to the hospital with the diagnosis of ST-segment elevation MI (STEMI) or non-ST-segment elevation MI (NSTEMI) between September 2016 and January 2020. Patients with a previous history of coronary artery bypass grafting (CABG) were not included in the study because the SYNTAX score could not be calculated. Patients with a myocardial infarction with non-obstructive coronary arteries (MINOCA) and patients with secondary troponin elevations without obstructive coronary lesions were not included in the study. Patients who did not undergo PCI and for whom CABG was chosen as the treatment were not included in the study. Patients who were not part of the follow-up were not included in the study. The inclusion and exclusion criteria for the patients are summarized in Figure 1.

STEMI was defined as ST-segment elevation in at least two consecutive leads on the electrocardiogram (ECG). NSTEMI was defined as an increase or decrease in cardiac troponin value without persistent ST-segment elevation on the ECG. All patients underwent coronary angiography (CAG) and PCI. An ECG was performed for all patients after admission to the emergency department, and acetylsalicylic acid was given at a loading dose of 300 mg after the first contact. All the patients were started by loading the appropriate dose of clopidogrel, prasugrel, or ticagrelor as the second antiplatelet agent based on their clinical and demographic conditions. In most patients, the procedures were performed using femoral access. In most STEMI patients, after the diagnosis, the emergency room-to-balloon time was kept below 10 min. In NSTEMI patients, CAG and PCI procedures were applied within the first 3–24 h after admission to the emergency department. In lesions with dense thrombus, thrombus aspiration or tirofiban was administered both intracoronally and by infusion. All the patients were monitored in the coronary intensive care unit for the first 24 h, with medical treatments applied to the patients as recommended by the guidelines. All the operators who performed the angiographic operations on the patients consist of operators who perform more than 250 CAG and PCI operations annually. Appropriate PCI was applied according to the characteristics of the coronary lesion in the patients. PCI was performed on the responsible lesion, and an intra-aortic balloon pump was used in patients with hemodynamic instability in the absence of response to inotropic and vasopressor treatments. While the procedure was performed after temporary pacemaker implantation in patients with inferior and complete AV block, adequate fluid resuscitation was performed in patients with right ventricular infarction. Considering the current clinical conditions of the patients and due to a lack of experience, routine intravascular ultrasonography (IVUS), optical coherence tomography (OCT), and fractional flow reserve (FFR) were not used. Pre-dilatation of the lesions, stent implantation, and post-dilatation procedures were applied to patients as appropriate.

Coronary flow with Thrombolysis in Myocardial Infarction (TIMI) 0–1 after PCI is recorded as a failed intervention/no-reflow. TIMI grade 0: there is no antegrade flow beyond the point of occlusion; grade 1: the contrast material passes across the obstruction and opacifies the entire coronary bed distal to the obstruction for the duration of the angiography; grade 2: partial perfusion contrast opacifies the coronary vessel to the distal but clearance is bad; and grade 3: normal and complete perfusion. Killip scores of the patients during hospitalization were taken from their files and noted. Killip 1 is normal, and Killip > 1 is noted as having varying degrees of cardiac dysfunction and pulmonary edema. Patients with cardiac arrest who underwent successful cardiopulmonary resuscitation at admission were included in the study. Cardiogenic shock patients who were hypotensive at admission and had hypotensive organ dysfunction were included in the study. 

The SYNTAX score of the patients was calculated angiographically by 2 experienced interventional cardiology specialists. While calculating the SYNTAX score, primarily right coronary dominance or left coronary dominance was determined. Lesions of vessels with a stenosis of more than 50% and a diameter of more than 1.5 mm were included in the calculation by examining each vessel and each lesion individually. In addition, whether the lesion is total occlusion or not, whether it has a blunt end or not, and whether there is antegrade collateral flow or not, were considered during scoring. Bifurcation lesions were classified according to the Medina classification, and scoring was calculated accordingly. Scores were given according to ostial lesions, whether the lesions were tortuous or contained thrombus. Afterward, these scores were summed, and the SYNTAX score was obtained. In the calculation of cul-SS, only the SYNTAX score of the lesion responsible for the infarct was calculated, and the scoring of other lesions and vessels was not included. Patients with coronary TIMI 0–1 flow were evaluated as having total occlusion. When calculating the SYNTAX score, lesions with a vessel diameter > 1.5 mm and a stenosis level above 50% were taken into consideration. Bifurcation lesions were evaluated as lesions requiring double wire and double stents and were included in the study [5,6,7].

Deaths in 30-days and major complications, such as stroke, contrast-induced nephropathy, stent removal, coronary rupture, coronary dissections with TIMI 0–1 flow, complications requiring intervention (femoral hematoma, femoral pseudoaneurysm), bleeding (pulmonary, gastrointestinal, or urinary hemorrhage) requiring treatment, resistant ventricular arrhythmias, and loss of major side branches (diameter > 1.5 mm), were accepted as primary outcomes. Contrast-induced nephropathy is defined by a rise in creatinine of 25% from baseline or 44 μmol/L within 48–72 h after administration of a contrast agent in the absence of any other explanation.

In statistical analysis, while analyzing the normality and variance of the parameters, the Kolmogorov-Smirnov test and the Shapiro-Wilk test were used. The chi-square test and Fisher’s exact test were used in the analysis of categorical variables; categorical variables were expressed in numbers and percentages. After that, the Mann-Whitney U test or Student’s *t* test was applied to the numerical variables, and the data were summarized as median and 25–75% interquartile range (IQR) or mean ± standard deviation. An ROC-curve analysis of SYNTAX scores and cul-SS was performed for primary outcomes. The data are summarized with area under the curve (AUC) values, 95% CIs, and *p* values. A logistic regression analysis was performed to determine the predictors of primary outcomes. First, a univariate logistic regression analysis was performed. Afterward, a multivariate logistic regression analysis was performed with variables with *p* < 0.1. Results are given with a 95% confidence interval, and *p* < 0.05 was accepted as the significance level. SPSS.22 and STATA.17 were used in the statistical analyses.

## 3. Results

There were 1284 patients in the presented study. The median age of the patients was 61 (53–70 IQR), and 931 (72.5%) of the patients were male. On admission, there were 651 (50.7%) patients with a diagnosis of STEMI, 37 (2.9%) with cardiac arrest, 20 (1.6%) with cardiogenic shock, and 55 (4.3%) with Killip class > 1. The SYNTAX score and cul-SS were 10.5 (7–16.5 IQR) and 8 (5–10 IQR), respectively. Most of the culprit vessels were 530 (41.3%) LAD, and the others were 420 (32.7%) right coronary artery (RCA), 323 (25.2%) circumflex artery (CX), and 11 (0.9%) LMCA. Drug-eluting stents (DES) were used in 1100 (85.7%) cases. Bifurcation lesions in the culprit vessels were observed in 77 (6%) patients, and failure of PCI or no-reflow was observed in 39 (3%) patients. Major complications were observed in 36 (2.8%) patients and death in 42 (3.3%) patients. Stent thrombosis was detected in 24 (1.9%) patients, and 78 (6.1%) patients had primary outcomes. The demographic features and angiographic findings of the patients are listed in Table 1.

Major complications included a ventricular tachycardia storm resistant to medical treatment in 1 patient (cul-SS: 11), hemorrhagic stroke in 2 patients (cul-SS: 9, 9.5), gastrointestinal bleeding requiring transfusion in 2 patients (cul-SS: 20.5, 8), a peripheral intervention site complication requiring surgical intervention in 3 patients (cul-SS: 6, 2, 9), pulmonary hemorrhage in 1 patient (cul-SS: 9), mitral chordae rupture in 1 patient (cul-SS: 7), coronary dissection in 7 patients (cul-SS: 10, 14.5, 2, 21, 11, 9, 11), medically resolved contrast-induced nephropathy (CIN) in 8 patients (cul-SS: 10, 17, 9, 7, 21,13 11.5, 7.5), CIN requiring hemodialysis in 5 patients (cul-SS: 9, 19.5, 13.5, 9, 14.5), loss of major coronary side branches in 2 patients (cul-SS: 11, 12), a coronary rupture in 2 patients (cul-SS: 21.5, 3.5), and ischemic stroke in 2 patients (cul-SS: 6, 14). All the major complications are presented in Table 2.

**Figure 2:**
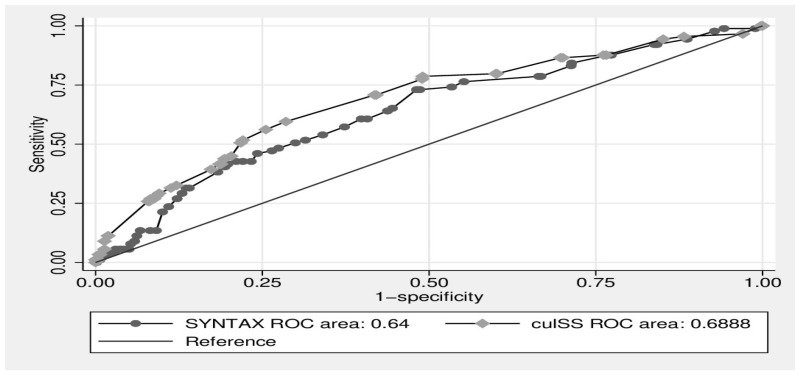
ROC-curve analysis of SYNTAX and cul-SS for primary outcomes.

An ROC analysis was performed to compare the predictive accuracy of SYNTAX and cul-SS for stent thrombosis and primary outcomes. The area under the curve (AUC) for SYNTAX and cul-SS for predicting stent thrombosis were 0.54 and 0.55 (*p* = 0.41, *p* = 0.40), respectively. An ROC analysis was performed to compare the accuracy of SYNTAX and cul-SS for the primary outcomes. The AUC for SYNTAX was 0.64 (95% CI: 0.579–0.700) and for cul-SS it was 0.68 (95% CI: 0.628–0.749), with a significant difference between the two ROC analyses (*p* = 0.026) (Figure 2).

To identify the predictors of primary outcomes, multivariate logistic regression analysis was performed using variables with a *p*-value < 0.1, such as age, STEMI on admission, cardiac arrest on admission, Killip class > 1, cardiogenic shock on admission, culprit LMCA lesion, culprit bifurcation lesion, SYNTAX score, and cul-SS. Age (OR: 1.03; 95% CI: 1.01–1.05; *p* < 0.001), STEMI on admission (OR: 1.63; CI: 1.03–2.56; *p* = 0.03), Killip class > 1 (OR: 12.3; CI: 5.79–26.1; *p* < 0.001), cardiogenic shock on admission (OR: 3.65; CI: 1.04–12.7; *p* = 0.04), and cul-SS (OR: 1.08; CI: 1.04–1.12; *p* < 0.001) were found to be associated with primary outcomes in multivariate logistic regression analysis. Regression analysis results are listed in Table 3.

## 4. Discussion

Our study is one of the first to show the prognostic value of the culprit-SYNTAX score system in patients with ACS. The culprit-SYNTAX score was found to be as successful as the SYNTAX score in predicting stent thrombosis and superior in predicting primary outcomes. From this point of view, it is possible to say that the power to predict the short-term prognosis in the hypothesis of our study is superior to the SYNTAX score. Our study is not the first to evaluate cul-SS. There is a study that retrospectively evaluated fewer patients presenting with cardiogenic shocks. The difference between that study and ours was that fewer patients were included in that study, and the patient population was limited to only cardiogenic shock patients. Since we included all ACS patients in our study, we have both conducted our study with a larger patient group and extended the power of our scoring system to ACS patients who met the study criteria in all possible clinical scenarios. In the study published by Kyehwan Kim et al. during the writing phase of our study, for the first time, the cul-SS was compared with the SYNTAX score and evaluated in cardiogenic shock patients presenting with STEMI. In that study, the cul-SS was found to be superior to the SYNTAX score in terms of the predictive power of in-hospital mortality rates [17]. The population of their study consisted of patients with myocardial infarction presenting with cardiogenic shock. In their study, the power of cul-SS to predict in-hospital mortality was found to be moderate. However, when they added the TIMI flow and the no-reflow phenomenon, they obtained a strong prediction value. According to the results of the multivariate regression analysis performed in our study, having a cardiogenic shock at the time of admission was found to be an independent predictive factor for primary outcomes. Additional independent predictors of primary outcomes include age, STEMI presentation, Killip class > 1, and cul-SS. Age, STEMI presentation, Killip class > 1, and cardiogenic shock on admission are well-known predictors of both short- and long-term mortality and morbidity [18,19,20]. In addition, cul-SS was found to be a short-term prognostic predictor.

Serhan Farhan et al. showed that LMCA and proximal LAD-localized lesions increase mortality as a result of their studies. LMCA and LAD proximal lesions have the highest score according to localization in calculating the SYNTAX score [15]. This situation causes both the SYNTAX score and cul-SS to be high in patients. In the regression analysis of our study results, although having a culprit LMCA lesion provided a significant prediction in the univariate analysis, this significance was lost in the multivariate analysis. While one of the reasons for this is the success of the procedures, it may also be that this patient group often presents with cardiogenic shock and pulmonary edema [21]. Since multivariate regression analysis was performed with these parameters, it may have lost its level of significance. Another reason may be the low number of patients with LMCA culprit lesions in the study group. As it is known, culprit LMCA lesions are often fatal. Some of these patients may die before they can be admitted to the hospital or diagnosed. In the study by Serhan Farhan et al., they did not evaluate whether the clinical outcome of patients was due to lesions or to cardiogenic shock conditions caused by these lesions. Therefore, the number of patients in this group may be small. In addition, the regression analysis results of culprit bifurcation (Medina 1.1.1) lesions were found to be similar to those of culprit LMCA lesions. While the univariate regression analysis was significant, it lost its significance in the multivariate regression analysis. This showed us that culprit bifurcation lesions are less important for prognosis than classical prognostic factors. Of course, the effect of successful bifurcation procedures performed here should not be ignored. Previously, the view of full revascularization was dominant in patients presenting with acute MI [22]. Although this approach was not wrong in selected patient groups, it was observed that there were some handicaps to using this treatment in the same way in all MI patients. When calculating the SYNTAX score, the scoring of lesions with anatomical complexity, tortuosity, and total stenosis is high, and this situation both decreases the success of the procedure, prolongs the procedure time, and increases the risk of cardiovascular complications such as the no-reflow phenomenon.

An unexpected result based on our study is that successful resuscitation was performed, but the patients presenting with cardiac arrest did not show its effect as an independent predictor in the multivariate regression analysis. As it is known as the underlying cause, if cardiac arrest due to coronary reasons is not witnessed, mortality rates are very high. Arrhythmic complications are often the cause of cardiac arrest in these patients. Early successful resuscitation responds well to early intervention for arrhythmic complications. Often, when these patients are brought to the hospital, they do not have time to perform primary PCI, and the patients are lost, or CAG is not applied to the patients due to the developing hypoxic situation. The situation here suggests that the group consisting of witnessed cardiac arrest events and even in-hospital cardiac arrest patients is in the majority. Accordingly, it may have influenced the primary outcomes in this way.

As it was mentioned above, the prolongation of the angiography procedure is a situation we do not want for these patients. Because of the longer processing time, a greater amount of opaque is used for image acquisition. This may lead to an increase in mortality because of acute renal failure and an increased need for dialysis after the procedure, especially in STEMI patients, since we do not know the basal renal values most of the time. In our study, no relationship was found with contrast-induced nephropathy, which may be due to the low number of patients who developed contrast-induced nephropathy. An evaluation should be made in patient groups with a higher risk of contrast-induced nephropathy. Similarly, the longer the procedure, the higher the risk of catheter-induced thrombosis, which can lead to a STEMI that leaves a defect in vital organs such as the brain. Therefore, it can be said that cul-SS is also associated with procedural complications in some cases, and the prognosis may be related to the vascular structure, the clinic, the procedure, and the material used in acute MI patient cases. The treatment method applied to the patient with the SYNTAX score (full revascularization or intervention of the culprit lesion) may be optimal in the presence of a suitable stent size, microcatheters, or special guidewires. Depending on the hospital’s potential, stent sizes, microcatheters, or special guidewires may not always be available. Therefore, the cul-SS can be an important guiding marker in such cases.

The SYNTAX score is a scoring system that calculates the total coronary plaque burden and determines the revascularization options for patients. In many subsequent studies, it has been evaluated whether it will be beneficial in terms of prognosis. The SYNTAX score has some limitations in clinical use, with STEMI patients being the best example in this regard; PCI is almost always applied to the totally occluded lesion in these patients, regardless of the SYNTAX score. Our aim in developing the cul-SS calculation was to obtain a short-term prediction of prognosis in ACS patients using this scoring system. For these reasons, we do not consider the cul-SS to be an alternative to the SYNTAX score, and we think that the intended use of both is different. Combined use, i.e., choosing the revascularization method based on the SYNTAX score and determining the risk of each intervention by calculating cul-SS during the PCI procedure, may be beneficial for both ACS and non-ACS patients in terms of determining the difficulty of the procedure and determining the short-term prognosis, but this requires further studies. The most important limitation in terms of using the cul-SS for the determination of long-term prognosis in patients with chronic coronary syndrome was that it reflected the myocardial tissue at risk in the hypothesis of the cul-SS. In the ISCHEMIA study, no superiority of invasive intervention to medical treatment in chronic coronary artery disease was demonstrated [23]. Based on this, we think that the cul-SS will have a limited contribution in terms of long-term prognosis, except to predict the risk of the procedure in patients with chronic coronary syndrome.

From this point of view, it is not wrong to think that the localization and anatomical complexity of the culprit lesions can both predict the myocardial tissue under threat and increase the rates of short-term death and complications by increasing the rate of procedure-related complications and failures. Accordingly, we think that this scoring system can be predictive of both the treatment and follow-up of patients in terms of short-term prognosis.

## 5. Conclusions

The results show that cul-SS appears to be as successful as the SYNTAX score in predicting stent thrombosis in patients with ACS and more successful in predicting 30-day deaths and major complications.

## Figures and Tables

**Table 1:** Demographic and angiographic parameters.

Parameters	Value
Gender (Male%)	931 (72.5%)
Clinic on admission (STEMI%)	651 (50.7%)
Cardiac arrest on admission	37 (2.9%)
Shock on admission	20 (1.6%)
Killip class > 1 on admission	55 (4.3%)
Culprit vessel	LAD: 530 (41.3%) CX: 323 (25.2%) RCA: 420 (32.7%) LMCA: 11 (0.9%)
Bifurcation lesion on culprit vessel	77 (6%)
Failure PCI and no-reflow (TIMI-0)	39 (3%)
Previous PCI	243 (18.9%)
Deaths	42 (3.3%)
Major complications	36 (2.8%)
Stent type (DES%)	1100 (85.7%)
Stent thrombosis	24 (1.9%)
Age	Median 61 (53–70 IQR)
SYNTAX	Median 10.50 (7–16.50 IQR)
Cul-SS	Median 8 (5–10 IQR)

Abbreviations: Cul-SS: culprit-SYNTAX score; CX: circumflex coronary artery; DES: drug-eluting stent; LAD: left anterior descending coronary artery; LMCA: left main coronary artery; PCI: percutaneous coronary intervention; RCA: right coronary artery; STEMI: ST-segment elevation myocardial infarction.

**Table 2:** Major complications.

Complications	Numbers	Cul-SS
Resistant ventricular tachycardia	1	11
Hemorrhagic stroke	2	9, 9.5
Gastrointestinal bleeding	2	20.5, 8
Peripheral artery complication	3	6, 2, 9
Pulmonary hemorrhage	1	9
Mitral chorda rupture	1	7
Coronary dissection	7	10, 14.5, 2, 21, 11, 9, 11
Contrast induced nephropathy resolved medically	8	10, 17, 9, 7, 21, 13, 11.5, 7.5
Contrast induced nephropathy requiring dialysis	5	9, 19.5, 13.5, 9, 14.5
Loss of major coronary side branches	2	11, 12
Coronary rupture	2	21.5, 3.5
Ischemic stroke	2	6, 14

**Table 3:** Univariate and multivariate logistic regression analysis for primary outcomes.

Parameters	Univariate Analysis			Multivariate Analysis		
	Odds Ratio (OR)	95% CI	*p* Value	OR	95% CI	*p* Value
Age	1.02	1.01–1.04	0.002	1.03	1.01–1.05	<0.001
Male gender	1.05	0.68–1.63	0.8	-	-	-
STEMI on admission	2.43	1.60–3.69	<0.001	1.63	1.03–2.56	0.03
Cardiac arrest on admission	14.59	7.39–28.82	<0.001	1.28	0.017–98.68	0.9
Killip class > 1	10.80	6.09–19.17	<0.001	12.30	5.79–26.12	<0.001
Cardiogenic shock on admission	4.63	1.74–12.30	0.002	3.65	1.04–12.77	0.04
Culprit LMCA lesion	7.05	2.08–23.86	0.002	1.62	0.32–8.13	0.5
Culprit bifurcation lesion	2.48	1.34–4.59	0.004	1.77	0.88–3.54	0.1
History of coronary artery disease	0.91	0.55–1.51	0.7	-	-	-
Bare metal stent	1.09	0.49–2.42	0.8	-	-	-
SYNTAX score	1.05	1.03–1.08	<0.001	0.99	0.95–1.03	0.8
Cul-SS	1.11	1.076–1.12	<0.001	1.08	1.04–1.12	<0.001

Abbreviations: CI: confidence interval; Cul-SS: culprit-SYNTAX score; LMCA: left main coronary artery; STEMI: ST-segment elevation myocardial infarction.

## Data Availability

Authors can make data available on a reasonable request through a data access committee, institutional review board, or the authors themselves.

## Abbreviations

ACS	Acute Coronary Syndrome
AUC	Area Under Curve
CABG	Coronary Artery Bypass Grafting
CAD	Coronary Artery Disease
CAG	Coronary Angiography
CI	Confidence Interval
CIN	Contrast-Induced Nephropathy
Cx	Circumflex Coronary Artery
CTO	Chronic Total Occlusion
Cul-SS	Culprit lesion’s SYNTAX Score
DES	Drug-Eluting Stent
ECG	Electrocardiogram
FFR	Fractional Flow Reserve
IQR	Interquartile Range
IVUS	IntraVascular UltraSonography
LAD	Left Anterior Descending Artery
LMCA	Left Main Coronary Artery
MI	Myocardial Infarction
MINOCA	Myocardial Infarction with Non-Obstructive Coronary Arteries
NSTEMI	Non-ST segment Elevation Myocardial Infarction
OCT	Optical Coherence Tomography
OR	Odds Ratio
PCI	Percutaneous Coronary Intervention
RCA	Right Coronary Artery
ROC	Reciever operating characteristic
STEMI	ST segment Elevation Myocardial Infarction
SYNTAX	SYNergy between Percutaneous Coronary Intervention with TAXus and Cardiac Surgery
TIMI	Thrombolysis In Myocardial Infarction
TLR	Target Lesion Revascularization
